# Thoughts on Fever

**Published:** 1846-06

**Authors:** James C. Harris

**Affiliations:** Wetumpka, Alabama


Art. II.—*Thoughts on Fever.* By James C. Harris, M.D., of Wetumpka, Alabama.

The following remarks on the character of the seasons and prevailing fevers of the past year are submitted under the conviction, that it is the duty of every physician to do something to elevate the character and increase the usefulness of his profession; and although they may contain but little, if anything, calculated to effect either object, still as a history of the past they may by comparison throw some light upon the coming events of the future. In entering upon the subject of fever I have a deep sense of the difficulty of my task. Its cause, the morbid phenomena attending it, the modus operandi of the poison producing it, and of the remedies by which it is treated, all are still involved in obscurity. Whether, in adding another to the many hypotheses which fever has called forth, I have added anything of value to the stock of medical doctrine, is left for others to decide.

Medicine of late, under the influence of the lights furnished by organic chemistry, together with those derived from the study of pathological anatomy, has been gradually advancing, and must continue to advance, aided by such powerful auxiliaries, until many of the present errors and abuses are reformed, and a more rational philosophy, and a better practice established upon their ruins. Should I fail in my desire of being useful in every other particular except only that of calling the attention, and fixing the thoughts of my bretheren upon the importance of the subjects involved in this essay, I shall consider myself amply repaid for the labor it has cost in its preparation.

That some of the doctrines advanced are not altogether without novelty, and will therefore by many be considered visionary and false, I entertain no doubt; neither have I the vanity to believe that they are placed by my arguments upon as unfailing a foundation as might be desirable, or as their importance demands; nevertheless I am as thoroughly impressed with their correctness as though they had received the finishing touch of a master hand. I shall therefore proceed, without further preface, to the consideration of atmospheric air—that immense mass of permanently elastic fluid, which surrounds our globe, and in which we are continually immersed; and which we are informed by chemistry consists essentially of oxygen and nitrogen in the proportion of 21 of the former, to 79 of the latter, with a small trace of carbonic acid, and an atmosphere of watery vapor. Chemistry farther teaches us that these proportions, at the top of our highest mountains and in the deepest vallies, at the pole, and under the equator, —in every situation and latitude, and amid all contending forces are unalterably maintained, an accumulation of one never taking place at any given point to the diminution of the other. True, the quantity of oxygen inspired differs with the temperature and density of the atmosphere, but its combining equivalent is always the same. Hence it is obvious that, in an equal number of respirations, we consume more oxygen at the level of the sea than on a mountain, in winter than in summer, at the north pole than under the equator; and that under all these varied circumstances we find it answering most perfectly the ends for which it was designed, and containing within itself one of the chief elements of organic life.

But whilst we cannot fail to be forcibly struck with the perfect power possessed by this great link in the chain of creation of maintaining its purity, still we must not forget that, owing to the great extent of vegetable and animal decomopsition perpetually in progress, on the surface of the earth, the atmosphere is necessarily adulterated by other gases known to be injurious to health and destructive of life. The conclusion from the researches which have been best conducted, I believe, is that the exhalations furnished from vegetable matter are the principal, if not the sole cause of our autumnal fevers. We are told, indeed, that the experiments of Broschi in the most pestilential regions of the Campagna di Roma, and those of Moschati on the air of some insalubrious rice fields in Tuscany, as well as those of Rigand de l'Isle in the marshes of Languedoc, all made with the view of detecting this poison, resulted in nothing more than the discovery of a few flocculi possessing the properties of animal matter. The scientific and laborious experiments instituted with the same view by Boussingault were attended with no better success. Still experience has established the existence of a subtle poison by its deleterious effects, and observation has shown that in its action upon the human system it bears a greater resemblance to carbonic acid than to anything else. Other gases, however, the result of other decompositions, are also found suspended in the atmosphere ready to do the work of destruction, but not in so great abundance, or of so deleterious a character. Some have also contended for the influence of barometrical and electrical states of the air; but how far they are concerned in the development of autumnal fever, I shall not at present stop to inquire.

To be convinced of the existence of a gaseous poison productive of fever, we have only to call to our aid the fact, that where ever we find a large amount of vegetable matter in connection with heat and moisture, there we meet with febrile diseases. To this effect we have the concurrent testimony of the profession everywhere. It is found to be true not less of the Ganges than of the Nile, of the Mississippi valley than of the Pontine marshes. A fervid sun and a fermenting mass of vegetable substances, are unfailing sources of bilious disorder. These positions may be considered unquestionable. The inquiry which then comes up is, how does this deleterious agent operate upon the living system?

That it acts mainly upon the blood in the lungs, through the medium of respiration, is rendered probable by the fact, that in all malarious districts and diseases, the blood appears to be imperfectly oxygenized, never exhibiting that high arterial color known to exist in good health. This black blood is known not to be the natural stimulus of the heart and arteries, upon the healthy performance of whose functions depends the health of the whole economy. Weakened action of the heart, and depressed and perverted nervous action, follow as a necessary consequence, first perceptible on the pulse, and the nerves of the cerebro-spinal axis, as is manifest from the dulness, headache, chilliness, &c.

Now as it is a well known and invariable law both of the nervous and muscular systems, that they must act and repose by turns; between these systems there is, moreover, so intimate a connection that during the different stages of febrile commotion all parts of the system participate more or less in the general disturbance. Congestion of the abdominal, thoracic, and cerebral viscera are first created. These being repeated, to be again followed by more violent reaction, local inflammation is at length set up in some one or more of the different organs. These now become a new source of irritation, which reacting upon existing derangements, continues to perpetuate the circle of morbid phenomena. But it must be borne in mind that it is only when the atmosphere is loaded with malaria, and when this poison is aided by aerial vicissitudes, and also by some imprudence on the part of the patient, that the morbid alterations to which the blood is subjected are adequate to the production of the varied phenomena of fever.

To recapitulate briefly: the positions assumed are—1st. That the remote cause of fever consists in an additional element in the air; and that it resembles more nearly carbonic acid in its effects upon the system, than any other known element. 2dly. That this poison in the atmosphere through the medium of respiration, the skin, and the stomach, produces an alteration in the blood, upon which deranged nervous action ensues, with local determinations and inflammations. All forms of malarious fever in their first stage, there is good reason to believe, are of a *general* character, and only become local from repeated reactions upon the organization.

Taking, then, as established the foregoing positions, I come next to the consideration of what I consider a more difficult part of the performance; to wit: the modus operandi of some of the leading articles usually employed in the cure of disease, and more particularly in the treatment of fever. This is a vexed question, respecting which all shades of opinion have been entertained, from that which refers the action of all medicinal agents to their admission into the blood, to that which denies to any foreign substance the power to reach the circulation in its formal state. This latter opinion, if it has not found as large a body of friends as the other, has secured the favor of some very able advocates, and has been defended with great eloquence and ingenuity. But it rests upon very insufficient evidence, and the progress of science renders its basis more insecure every day. It cannot be considered questionable, that a great number of medicines enter the bloodvessels, and find their way to every part of the body. It is through this avenue that many of our remedies reach the seat of morbid action. Quinine, for instance, when swallowed and mixed with the contents of the stomach, passes directly into the upper intestines; where it is taken up by the lacteal and absorbent vessels, and delivered under the influence of the vital forces, essentially quinine, into the circulation. Upon this subject M. Piorry remarks, that in twenty or twenty-five minutes after a person has taken sulphate of quinine the urine tastes strongly bitter, and that he gave a quantity of urine of this kind to Vallée to analyze, who obtained from it sulphate of quinine in crystals. Landerer, an apothecary of Athens, obtained a similar result from the urine of a patient affected with intermittent fever, who had taken in some paroxysms 40 grains and even a drachm of the muriate of quinine. Pereira, (Mat. Med., vol. 2, p. 456,) also proposes, upon the authority of some other writer, the cure of intermittent fever in the child by its administration to the mother during the months of lactation. From all of these facts it is as evident that quinine is absorbed and carried into the circulation as that it is swallowed. Of this there cannot be a doubt. How then does it act after having become mingled with the circulating fluids? The following explanation is submitted: The quinine having come in contact with the malarious poison in the round of the circulation, neutralizes and destroys it; thereby the blood is improved, receives oxygen more readily, becomes more perfectly arterialized, imparts, consequently, more force to the nervous centres, and increased energy to all the vital functions. That such is the case the recorded experience of a large majority of the profession goes strongly to prove. Hence the fact that in those depressed states of the system which occur in congestive chill, quinine may be given with safety in doses which in another condition of the system would give rise to distressing effects. In the morbid state attendant upon the chill, the principal force of the medicine is expended upon the malarious poison in the blood, neutralizing and correcting it, and thereby elevating the vital energies; but in an opposite state it acts upon the blood entirely, giving to it an additional power to absorb oxygen, and thereby adding fuel to an already consuming fire.

It is also probable that an additional property is communicated to the venous blood by the quinine before it enters the lungs, by virtue of which it acquires the power to elevate our temperature, and increase the activity of the heart. That this is the fact, to some extent, all the attendant circumstances seem to indicate.

The following facts bear upon this point, and appear to me to sustain the view I entertain of the modus operandi of quinine. Professor Yandell, in his Prize Essay on Bilious Fever, remarks, that a patient of his, "a young man from Alabama, having had a severe attack of pleurisy in the winter, fell into chills and fever in the spring. He tried the quinine in doses of one and two grains, and with them succeeded in checking the disease; but it still returned. In one of the paroxysms, perhaps the second or third of a relapse, I gave him 16 grains at a single dose, in the height of the chill. Warmth was soon restored; sweating came on without any well marked febrile paroxysm; and the chills left him to return no more." From the operation of the remedy in this and analogous cases Professor Yandell concludes that quinine, in small doses, stimulates, and in large ones calms and subdues nervous and arterial excitement.

Dr. Byrne in an analysis of a Report to the Surgeon General, on large doses of quinine (Western Journal of Med. and Surg. for 1845, p. 250,) says, that the terms tonic, stimulant, or sedative convey no just impression of the effects of quinine, its whole force being expended upon the nervous system, and its influence over the circulation being doubtful and uncertain. The effects perceived, he continues, are those of an anti-periodic, or in more comprehensive phrase, a direct antidote to the malarial poison. How this effect is produced, he adds, must remain unanswered.

Now the explanation we would give of the action of this remedy is, that it is always *stimulant*, and that its peculiar effect upon the skin, the circulation, and the nervous system depends upon their state at the time of its administration. Its uniform tendency is to destroy the fever-poison where-ever found, in the lungs, the venous trunks, or the capillaries.

That the result of an overdose might be the reduction of a full, strong pulse, and increased nervous action, I do not pretend to deny; but that this is not by any means the invariable result of an ordinary dose in similar stages of the system, any one may satisfy himself by an experiment such as I witnessed last summer. I witnessed the administration of quinine in 10 grain doses at intervals of an hour until 40 grains were taken, in stages of open excitement, with furred tongue, hot skin, and full, strong pulse, with no other effect than an aggravation of all the symptoms, and the addition of *tinnitus aurium*, and pain in the head. The reason of all this is obvious; the blood being sufficiently arterialized, the quinine did nothing more than add to the general excitement and distress, by virtue of its power manifested in the way heretofore described.

In the stage of collapse, or where there appears on the part of the system a tendency to sink, I highly approve of the liberal administration of quinine; it may be used also with safety and advantage, as all the world knows, on the decline of fever, or as soon as any softness of the pulse and moisture of the skin make their appearance. But that much harm has been done by its administration during stages of excitement, I am constrained to believe; and I trust the day is not far distant when a more rational view will be taken of the remedythan that which now leads to its employment in such opposite states of the system.

I will now offer a few remarks on the operation of *calomel*, and then dismiss this part of my subject. That this article produces its remedial effect chiefly by entering the circulation, is quite as certain as that quinine acts in that way, but here the analogy between the remedies almost entirely ceases. Calomel, owing to the peculiar and powerful influence it is known to exert over the liver and glandular system generally, is an article unique in its modus operandi. That it not only enters the circulation, but also may remain dormant in the system for several years, and then manifest some of its peculiar effects upon the glands of the throat and mouth, and upon the mucous membranes, in a very insidious and fatal manner, there is good reason for believing. The case of ulcerated tongue and mouth communicated by the writer in the April number of this Journal, (1842, page 319,) is one in point. But these truly interesting and singular effects occuring out of the usual course of its operation, once perhaps in ten thousand times, are to be ascribed rather to peculiarities of constitution, than to any inherent property in the remedy itself. That calomel also possesses the power to resist the influence of the vital forces, in a very eminent degree, is evident from the fact, that a gold watch worn next the skin, or gold rings upon the fingers during a mercurial saturation, become whitened with an amalgam of mercury. In its action in the removal of fever it seems to pervade the whole sanguiferous and glandular systems, imparting to the former certain powers that enable it to place the secreting apparatus of the latter in active operation. The circle of its action appears to be extended and powerful, no tissue of the system escaping its influence; and it is not asserting more than experience sanctions, when I say that a remedy with its stimulating and alterative properties, under judicious management, and on correct principles, is susceptible of almost universal application, and may fulfill almost any indication in the cure of disease.

When under the influence of this remedy the nausea and desire to evacuate the bowels by purging are to a considerable extent, I apprehend, brought about by certain specific and stimulating properties being added to the bile, and other secretions.

Without further preliminary remarks I will now proceed to the main object of this paper, which is to give some account of a fever with which Wetumpka was visited last autumn, and of the medical topography of the locality in which it prevailed.

The Coosa river, which separates East and West Wetumpka, after it enters the limits of the town, runs for the first half mile in nearly a south-west course, when it passes under the bridge, and then shifts its direction more to the south-east. Pursuing this course for about a mile more, and then turning west, it runs in a devious line until its name and waters are lost in those of the Tallapoosa.

In the original plan of the city, the streets were laid out North and South, East and West, crossing each other at right angles; but owing to the peculiar structure of the surface of the earth upon the eastern side this arrangement has been lost. Here Main street runs south from the end of the bridge, and Bridge street east, two hundred yards to the market house; forming an angle here upon its outer section with Gay street, then runs north-east and parallel with the river, to the north eastern extremity of the corporation.

At the north-eastern extremity of the city, and upon the eastern side of the river a range of hills sets in, at the distance of a quarter of a mile from the river, and continues parallel with it, gradually approaching it, until it arrives at the market house, where the range suddenly changes its direction due south, and continues this course until the hills are lost in the high lands separating the waters of the Coosa from those of the Tallapoosa rivers.

At low tides the current of the river above the bridge is thrown almost entirely against the western bank, leaving exposed to the action of the sun a large amount of decaying vegetable matter. There are also interspersed among the rocks on the shoals many pools of stagnant water, in which a mass of organic matter is continually decaying. One in particular commencing not far above the bridge, and extending some hundred yards up the river, is a fruitful source of exhalation. A large ravine also extends from the water's edge in the rear of the buildings on Bridge street nearly up to the market house, and receives the filth from the greater portion of this part of the city. There are other sources of fever of a local character upon both sides of the river; but as they are similar to those already described, I pass them by without any further notice, referring for a fuller topography to an article by the writer *on Diarrhœa*, published in this Journal in September, 1845, on pages 191–'2.

Now if the wind during the months of August and September sets in, and continues to blow regularly from the north and north-west, for three or four days in succession, over these prolific sources of malaria, what else could be expected, but that all the inhabitants residing on Bridge, Main, Gay, and Company streets, and the sides and tops of the adjacent hills, should become liable in a peculiar manner to fevers? And in the assertion that they always do suffer, to a greater or less extent, I am borne out by the observations of all who have paid any attention to the matter.

The summer and autumn of the past year were seasons as remarkable for their heat and dryness in this section of country, as perhaps any that have been experienced in many years. The thermometer stood steadily throughout the greater portion of the month of July at 4 o'clock, P.M., above

90°. On the 24th it stood at 102°—the highest point to which it was ever known to rise in this city. The dryness was as excessive as the heat, destroying almost entirely the crops of the planter. In fact all animated creation seemed to droop under the influence of the parched and heated atmosphere. Still the city remained comparatively free from disease of all kinds until about the 20th of July. At this time a few scattering cases of fever began to make their appearance. They were at first mild, and, so far as they fell under my observation, of an intermittent and remittent type, most generally the latter, attended with furred tongue, sick stomach, pain in the head, loins, and extremities, restlessness and dry, hot skin. As the season advanced, the pain in the head, extending through from temple to temple, became more frequent, and at times was of so violent a character as to give rise to delirium.

In one of these cases there was a peculiar tremor of the tongue upon protrusion; the tongue was covered with a dark brown fur.

The bowels also became painful, and tender to the touch, and manifested in a few cases a strong tendency to diarrhœa, the discharges being watery.

The heat of the surface was more pungent and intense than I remember to have remarked it since the fall of 1834. The restlessness, in such cases, was extreme and distressing. The pulse varied according to the stage of the case, and the hour of the day and night, being generally lowest about midnight, gradually augmenting in frequency and force until after the middle of the afternoon of the next day. It then gradually commenced declining, but scarcely ever coming down to the healthy standard; on the contrary, it averaged, the most of the time, from 120 to 130 pulsations in the minute, and sometimes even rose above this.

From the above brief description it will appear that the general character of the epidemic was a bilious remitting fever. The remissions were at no time very strongly or clearly marked; the disease was attended with local determinations and inflammations, most generally the brain or bowels being the suffering organs, and sometimes both.

In the treatment of the fever, in the early stages, the lancet, the cold and warm baths, and calomel and ipecac., in combination, were the means principally relied upon by myself for the reduction of excitement, and the preservation of the integrity of the various organs. In the more advanced stages, where the disease had been neglected, or the vital energies appeared overpowered or exhausted, calomel and quinine internally, with sinapisms and blisters to the extremities, and over the seat of the suffering organs, constituted the outline of my curative endeavors.

It was found at all times exceedingly difficult to conform the use of the quinine to the remissions, and on this account I often failed to derive any benefit whatever from its employment.

For the restlessness and watchfulness, and where there was a disposition in the bowels to serous passages, morphia alone, or in combination with calomel, was generally used, and found to answer the end desired. The greatest difficulty, however, experienced in the successful management of the disease, was to procure and keep up free, consistent bilious evacuations. This could only be done, in some cases, by the liberal administration of mercurial medicines; and to show the extent to which it sometimes became necessary to carry this practice, I will conclude this already too much extended paper, by extracting the following case from my note-book.

September 13, 1845. Called at midnight to see Mr. Lyon in consultation with Dr. A. N. Lightfoot, an intelligent and respectable practitioner of our city. The patient had been attacked on the first of the month with the usual symptoms of this fever. Upon my arrival Dr. L. informed me that he had bled the patient twice, blistered his wrists and epigastrium, and purged him liberally with calomel. The peculiarities in his case, were, an intermitting pulse, extreme gastric irritability, a strong disposition to watery discharges under the use of cathartics. His other symptoms were great heat, but no tenderness at the epigastrium, tongue coated with a dark brown fur, and no appearance of ptyalism, although he had taken large doses of calomel.

Under a joint prescription, he continued to take from 40 to 60 grains of calomel daily, for five or six days, in combination with an occasional dose of morphia. Upon this course, bilious, consistent discharges were soon produced, and easily kept up; and Mr. L., without experiencing any bad effects at the time upon the glands of his mouth, or in any other way from the medicine, is now in the full enjoyment of his usually good health.

March, 1846.

